# Concise Review: Getting to the Core of Inherited Bone Marrow Failures

**DOI:** 10.1002/stem.2543

**Published:** 2016-12-04

**Authors:** Soheir Adam, Dario Melguizo Sanchis, Ghada El‐Kamah, Sujith Samarasinghe, Sameer Alharthi, Lyle Armstrong, Majlinda Lako

**Affiliations:** ^1^ Department of MedicineDuke University Medical CenterDurhamNorth CarolinaUSA; ^2^ Hematology DepartmentMedical School, King Abdulaziz UniversityJeddahKSA; ^3^ Institute of Genetic Medicine, Newcastle UniversityUnited Kingdom; ^4^ Division of Human Genetics & Genome ResearchNational Research CenterCairoEgypt; ^5^ Department of HematologyGreat Ormond Street Hospital for Children NHS Foundation TrustLondonUnited Kingdom; ^6^ Princess Al Jawhara Al‐Brahim Center of Excellence in Research of Hereditary Disorders, King Abdulaziz UniversityKSA

**Keywords:** Inherited bone marrow failures, Human embryonic stem cells, Human induced pluripotent stem cells, Animal models

## Abstract

Bone marrow failure syndromes (BMFS) are a group of disorders with complex pathophysiology characterized by a common phenotype of peripheral cytopenia and/or hypoplastic bone marrow. Understanding genetic factors contributing to the pathophysiology of BMFS has enabled the identification of causative genes and development of diagnostic tests. To date more than 40 mutations in genes involved in maintenance of genomic stability, DNA repair, ribosome and telomere biology have been identified. In addition, pathophysiological studies have provided insights into several biological pathways leading to the characterization of genotype/phenotype correlations as well as the development of diagnostic approaches and management strategies. Recent developments in bone marrow transplant techniques and the choice of conditioning regimens have helped improve transplant outcomes. However, current morbidity and mortality remain unacceptable underlining the need for further research in this area. Studies in mice have largely been unable to mimic disease phenotype in humans due to difficulties in fully replicating the human mutations and the differences between mouse and human cells with regard to telomere length regulation, processing of reactive oxygen species and lifespan. Recent advances in induced pluripotency have provided novel insights into disease pathogenesis and have generated excellent platforms for identifying signaling pathways and functional mapping of haplo‐insufficient genes involved in large‐scale chromosomal deletions–associated disorders. In this review, we have summarized the current state of knowledge in the field of BMFS with specific focus on modeling the inherited forms and how to best utilize these models for the development of targeted therapies. Stem Cells *2017;35:284–298*

**Significance Statement**

Bone marrow failure syndromes are characterized by a common phenotype of peripheral cytopenia and/or hypoplastic bone marrow. Great strides have been made in the last 20 years both scientifically and clinically resulting in identification of more than 40 causative genes and improved transplant outcomes. In this review, we summarize the most recent findings achieved through application of animal models and stem cells which have generated important insight for disease physiopathology and improved patient care.

## Introduction

Bone marrow failure syndromes (BMFS) are rare diseases characterized by peripheral cytopenias and/or hypoplastic bone marrow and can either be inherited or acquired 1, 2, 3. The purpose of this review is to discuss the methods by which we may create in vitro models of these conditions, therefore we will focus upon inherited BMFS since although it is possible to induce bone marrow failure in experimental animals by administration of specific chemicals 1, the development of methods to replicate the phenotypes of acquired syndromes using cellular models is currently difficult. In essence, two broad mechanisms may be used to generate useful models of these diseases. Animal models can work well in cases where the genetic causes of the disease are known at the level of DNA sequence since it is feasible to engineer experimental animals in which the relevant mutations play analogous role to their human homolog. A significant problem with this approach is that the physiology of experimental animal species is often a poor match to that of humans and genes known to carry disease causing mutations in humans may not always have exactly the same mechanistic role in animals such as mice or rats. An increasingly attractive alternative is the use of pluripotent stem cell technology to create in vitro models of disease. Typically, this will involve “reprogramming” patient somatic cells followed by differentiation to the types of cells most affected by the disease. The behavior of these cells in vitro may replicate many features of the disease making them a valuable tool for increasing our understanding and identifying potential treatments. We will discuss both of these modeling options in turn.

BMFS have a broad clinical spectrum, sharing the failure of hematopoietic stem cells (HSCs) to produce functional blood cells and can affect patients of all ages 1, 2, 3. More than 30 inherited BMFS have been described and although they are generally rare, conditions such as Fanconi anemia (FA), dyskeratosis congenita (DKC), Diamond‐Blackfan anemia (DBA), Shwachman‐Diamond syndrome (SDS), congenital amegakaryocytic thrombocytopenia (CAMT), severe congenital neutropenia (SCN) and thrombocytopenia absent radii (TAR) are among the most common types. To date, more than 40 mutations in genes involved in maintenance of genomic stability, DNA repair and telomere biology have been identified in inherited BMFS. In addition, pathophysiological studies have provided insights into several biological pathways unraveling genotype/phenotype correlations, diagnostic approaches, and management strategies 1. Given the association between BMFS and genes involved in DNA repair mechanisms, it is perhaps unsurprising that many of the BMFS have a high predisposition toward malignancy 4, 5. FA, DKC, SDS, and CAMT often present with aplastic anemia and may evolve into myelodysplastic syndrome (MDS) and acute myeloid leukemia (AML). DBA, SCN, and TAR present with single cytopenia that rarely become aplastic but have increased risks of leukemia. Solid tumors like head and neck and anogenital squamous cell carcinoma are associated with FA and DC and osteogenic sarcoma with DBA 4, 5. A summary of the clinical features of these diseases together with associated mutations and therapeutic options is given in Table 1 6, 7, 8, 9, 10, 11, 12, 13, 14, 15, 16, 17, 18, 19, 20, 21, 22, 23.

**Table 1:** A summary of clinical features of IBMF together with causative genes and current therapies

**Bone marrow failure syndrome**	**Hematopoietic abnormalities**	**Other clinical features**	**Genes identified**	**Biological features**	**Disease management and treatment**	**References**
FA: genomic instability disorder caused by alterations in genes involved in replication‐dependant‐repair and removal of DNA crosslinks	Pancytopenia and bone marrow failure	Congenital abnormalities, growth retardation, bone marrow failure, increased risk of hematological malignancies and other solid tumors (mostly head and neck), radiosensitivity and premature ageing	*FANCA, FANCB, FANCC, FANCD1, FANCD2, FANCE, FANCF, FANCG, FANCI, FANCJ, FANCL, FANCM, FANCN, FACNP, FANCO, FANCS*	Hypersensitivity to DNA cross‐linking agents/intolerance to oxidative stress and frequent chromosomal aberrations pointing to a DNA damage response defect	Hematologic monitoring and solid tumor surveillance, androgen therapy, antioxidants, G‐CSF combined with Epo and/or androgens, allogeneic HSCT	6, 7, 8, 9, 10, 11, 12
DKC: multisystem disorder caused by defective telomere maintenance and/or ribosome function	Pancytopenia and bone marrow failure	Solid tumors (head, neck and colorectal), abnormal skin pigmentation, nail dystrophy, mucosal leucoplakia, periodontal disease, premature graying, osteoporosis, mental retardation, and pulmonary disease	*DKC1, TERT, TR, TINF2, RTEL1, NOP10, NHP2, WRAP53, C16orf57 and CTC1*	Accelerated telomere shortening in all leukocyte subsets resulting in cell loss or dysfunction and genomic instability	Hematologic monitoring and solid tumor surveillance, androgen therapy, G‐CSF combined with Epo, allogeneic HSCT	13, 14
DBA: selective reduction in erythroid precursors with macrocytic anemia	Red blood cell aplasia: macrocytic anemia, reticulocytopenia, and nearly absent erythroid progenitors in the bone marrow	Craniofacial, skeletal, cardiac and/or genitourinary abnormalities	*RPS24, RPS17, RPS7, RPS10, RPS19, RPS26, RPS27, RPS29, RPL5, RPL11, RPL26, RPL15, RPL27, RPL35*	Defective ribosome synthesis	Steroid therapy, red cell transfusion, iron chelation allogeneic HSCT	15
SDS: exocrine pancreatic insufficiency and hematologic abnormalities	Bone marrow failure, neutropenia, anemia, pancytopenia, MDS and leukemia	Exocrine pancreatic insufficiency, short stature, metaphyseal dysostosis, rib and thoracic cage abnormalities	*SBDS*	Hematopoietic progenitors have faulty proliferative properties and increased apoptosis linked to hyper activation of the Fas signaling pathway	Transfusions, pancreatic enzymes, antibiotics, G‐CSF, HSCT	16
CAMT: isolated thrombocytopenia and megakaryocytopenia with no physical anomalies	Thrombocytopenia with reduced or absent megakaryocytes in the marrow progression to pancytopenia and marrow hypoplasia can occur	No specific somatic abnormalities	*MPL*	Isolated thrombocytopenia and absence of megakaryocytes in the bone marrow caused by a defective response to TPO	Platelet transfusion, and antifibrinolytic agents for bleeding red cell transfusion for anemia, allogeneic HSCT	17, 18
SCN: very low neutrophil count often less than 0.5 × 10^9^/L	Severe neutropenia, myeloid series maturation arrest, progression to MDS and AML may occur in some patients		*ELA2, HAX1, AK2, GFI1, WASP, CSF3R, G6PC3, GATA1, JAGN1, VPS45*	Maturation arrest of granulopoiesis at the level of promyelocytes with peripheral blood absolute neutrophil counts below 0.5 x 10^9^/l and early onset of severe bacterial infections	G‐CSF, allogeneic HSCT	19, 20
TAR: newborns present with thrombocytopenia	Reduction in the number of platelets, reduction in number of megakaryocytes in bone marrow frequent bleeding episodes in the first year of life that diminish in frequency and severity with age	Characteristic bilateral absent radii (unilateral in ∼ 2%), with abnormal but present thumbs, facial dysmorphism, cardiac defects, and genitourinary malformations	*RBM8A*	Abnormal differentiation mechanism of megakaryocyte and platelet production	Platelet transfusions	21
RS: amegakaryocytic thrombocytopenia	Thrombocytopenia or AA	Limited pronation/supination of the arms due to proximal radioulnar synostosis	*HOXA11*	Certain cytokines stimulate the maturation of megakaryocytic progenitor cells, other signals as PF4, CXCL5, CXCL7 & CCL5 inhibit platelet formation	Supportive transfusions and allogeneic HSCT	22
Pearson Syndrome	Transfusion dependant macrocytic anemia, variable neutropenia and thrombocytopenia vacuoles in marrow precursors, ringed sideroblasts, anemia	Exocrine pancreas, liver, and renal tubular defects	Contiguous gene deletion/duplication syndrome involving several mtDNA genes	Mitochondrial DNA abnormalities	Supportive transfusions with blood and platelets as needed, treatment with Epo and G‐CSF for severe neutropenia	23

Abbreviations: AA, aplastic anemia; AML, acute myeloid leukemia; CAMT, congenital amegakaryocytic thrombocytopenia; DBA, Diamond‐Blackfan anemia; DKC, dyskeratosis congenita; Epo, erythropoietin; FA, Fanconi anemia; G‐CSF, granulocyte colony stimulating factor; HSCT, hematopoietic stem cell transplantation; IST, immunosuppressive therapies; MDS, myelodysplastic syndrome; SCN, severe congenital neutropenia; SDS, Shwachman‐Diamond syndrome; TAR, thrombocytopenia absent radii; TPO, thrombopoietin; IBMF, inherited bone marrow failures.

Although rare, the clinical impact of BMFS is undoubtedly significant. Experimental approaches to increase our understanding of these disorders are thus essential. Moreover, since many of the causative genes play important roles in the development and maintenance of the hematopoietic system, studying their dysfunctions may provide further insights into the production mechanisms of blood and immune cells. Thus, investigations using animal and cellular models of the group of diseases reviewed herein are of great value.

## Animal Models of the Bone Marrow Failure Syndromes Recapitulate Only Some Disease Features

Several strategies may be applied to the generation of animal models of BMFS with murine models being most typically applied. Genetically modified mice can be generated by either direct pronuclear injection of exogenous DNA into fertilized zygotes or injection of genetically‐modified murine embryonic stem cells (ESC) into a blastocyst. Direct pronuclear injection is technically demanding and often results in multiple, random integrations of the injected DNA into the genome, and the resulting disease phenotypes can vary depending on the expression level of the injected transgene. Mouse ESCs have the advantage that they can be genetically modified by means of homologous recombination, a process by which a fragment of genomic DNA introduced into a mammalian cell can recombine with the endogenous homologous sequence. This process is known as “gene targeting.” When such genetically modified ES cells are introduced into a preimplantation embryo, they can contribute to all cell lineages of the resulting chimeric animal. If this contribution also comprises germ cells and the chimeras are capable of breeding, it is possible to establish lines of animals that are both heterozygous and homozygous for the genetic alteration introduced into the ESCs. This process can be used to add DNA sequences to specific genomic loci (knock‐ins), a protocol most often used to generate cell lines with gene‐specific reporter systems 24, or to create point mutations with pinpoint accuracy. The technology for generating mouse models using gene targeting is well developed and while discussion of this technology is outside the scope of this article, excellent reviews on the subject are available 25, 26, 27.

Gene editing of murine ESCs has been used to create a number of genetically modified animals carrying mutations associated with some of the more common forms of BMFS. Despite the effectiveness of gene editing, these models do not always demonstrate all the mechanistic or symptomatic problems shown by humans. However, they have generated useful data on the mechanisms of BMFS. An overview of animal models created to date is provided in Table 2 28, 29, 30, 31, 32, 33, 34, 35, 36, 37, 38, 39, 40, 41, 42, 43, 44, 45, 46, 47, 48, 49, 50, 51, 52, 53, 54, 55, 56, 57, 58, 59. We hereby discuss individual examples of this approach:

### Fanconi Anemia

The hallmark of FA is hypersensitivity to DNA cross‐linking agents, intolerance to oxidative stress and frequent chromosomal aberrations pointing to a DNA damage response defect, all of which are used as diagnostic tests. To date 17 complementation groups have been identified and the genes encoding these groups A (*FANCA*), B (*FANCB*), C (*FANCC*), D1 (*FANCD1/BRCA2*), D2 (*FANCD2*), E (*FANCE*), F(*FANCF*), G (*FANCG*), I (*FANCI/KIAA1794*), J (*FANCJ/BRIP1*), L (*FANCL*), M (*FANCM*), N (*FANCN/PALB2*), P (*FANCP/SLX4/BTBD12*), O (*FANCO/RAD51C*), S (*FANCS*/*BRCA1*), and T (*FANCT/UBE2T*) have been cloned 6, 7, 8, 9, 10, 11, 12, 60. The FA core complex (FANCA, B, C, E, F, G, L, and M), functions as an E3 monoubiquitin ligase that responds to DNA damage or replication stress. The FA core complex ubiquitinates FANCD2 and FANCI (the ID complex) which then recruits FAN1 nuclease to help process and cleave the DNA damage, followed by further recruitment of FANCD1/BRCA2, FANCJ and FANCN to sites of DNA damage. Once the mono‐ubiquitinated FANCD2 and its associated partners (FANCI, FANCD1, FANCJ, and FANCN) are correctly localized on sites of DNA double strand breaks, they associate with other DNA repair and checkpoint proteins such as NBS1, ATR, CHK1 and CHK2, γ‐H2AX, RAD51, and BRCA1 to initiate DNA repair and arrest the cell cycle 4, 5. FANC proteins also unwind DNA triplexes, remodel DNA structures such as Holliday junctions, D‐loops and replication forks and channel the DNA lesions toward error free DNA repair achieved by homologous recombination.

Targeted single deletions in mouse of various genes such as *Fanca, Fancc, Fancd2,* and *Fancg* exhibit decreased long term HSC repopulating activity and germ cell loss in addition to cellular sensitivity to DNA interstrand crosslinks and oxidative stress, but lack the clinical characteristic of FA including marrow aplasia, hematological abnormalities, and early life tumorigenesis 28, 29, 30, 31, 32, 33. Cells cultured from all FA mouse models show accumulation of chromosomal aberrations when exposed to DNA cross‐linking agents, suggesting some degree of functional conservation of the FA DNA repair pathway between species. Cells present in the spleens of the mutant mice are highly susceptible to accumulation of unrepaired chromosomal aberrations following exposure to DNA cross‐linking agents and abnormal sensitivity to IFNγ. Moreover, *Fancc^‐/‐^* mice are particularly sensitive to the action of the DNA cross‐linking agent, Mitomycin C, administration of which causes bone marrow failure within 3‐8 weeks. A key inference from these data is that loss of function mutations of single genes of the FA pathway in mice do not compromise short‐term survival but rather restrict the capacity of mice to repair damage induced by environmental insults or DNA damaging agents. This implies that loss of additional genes might be needed to recapitulate the characteristics of human FA. Thus, several double mutant mouse models have been created to analyze processes that may enhance the development of FA. This approach is exemplified by the observation that while *Fancc^‐/‐^* mice do not develop bone marrow hypocellularity, the *Fancc^‐/‐^* and *Sod1^‐/‐^* double mutants develop this feature and go on to develop anemia and leucopenia, providing some evidence that oxidative stress contributes to bone marrow failure in FA 61. More recently, double mutants of *Fancd2^‐/‐^* and *Aldh2^‐/‐^* have been generated and these exhibit unusual sensitivity to endogenous aldehydes in utero 34, 35. Ethanol (a source of exogenous of acetyldehyde) exposure by postnatal double‐deficient mice rapidly precipitates BMFS and results in spontaneous development of acute leukemia, suggesting that the FA pathway counteracts acetaldehyde induced toxicity. Other promising models include the *Btbd12* knockout mouse, the ortholog of *Slx4* (*Fancp*) which mimics many features of FA including peripheral cytopenia, reduced fertility, dysmorphic features, ocular abnormalities, hydrocephalus, chromosomal instability, accumulation of damaged chromosomes, hypersensitivity to DNA crosslinking agents and abnormal lymphopoeisis 40. Whilst the data generated from such models are interesting, the need to create double knockouts to recapitulate, even in part the phenotype which in humans results solely from mutations in the *FANC* genes remains a significant problem. The potential greater susceptibility of mice to sustain and retain DNA damage and/or the presence of alternate regulatory mechanisms for FANC proteins in humans, indicate that murine FA models may not be optimal tools to understand the pathophysiology of FA and develop novel treatments. Furthermore, the nature of mutations in various types of FA is extremely heterogeneous, including point mutations, small insertions/deletions, splicing mutations, and large intragenic deletions, which makes it difficult to replicate exactly all human mutations through targeted gene knock‐ins/outs in the mouse system.

### Dyskeratosis Congenita

DKC is the first disorder to be etiologically linked to mutations in the telomere pathway 62. About 70% of DKC patients have identifiable germ‐line mutations affecting genes responsible for regulation and maintenance of telomeres 2. To date, nine genes have been associated with DKC phenotype; *DKC1, TERT, TERC, TINF2, WRAP 53, NOP10, NHP2, CTC1,* and *RTEL1* 63, 64. Two categories of mutations are found in DKC: mutations that decrease telomerase activity including those affecting Dyskerin (*DKC1*), *TERC* and *TERT*, and mutations that impair telomerase recruitment in genes such as *TIN2* 3. The resultant telomere shortening leads to cell senescence and stem cell exhaustion. Moreover, telomere attrition results in chromosomal fusion and genetic instability, which is at the root of development of secondary malignancies in affected individuals 4.

Gene editing of murine ESC has been used to model loss of function for most of these genes; however concerns about differences in telomere maintenance between mouse and human can limit the utility of these models. Although the telomeric DNA sequence is identical in both species, abnormalities in telomere maintenance and in telomerase function do not coincide in phenotype in humans and mice. Most strains of laboratory bred mice have telomeres 5‐10 times longer than humans, whereas absence of telomerase activity is only phenotypically present over several generations in mice and even heterozygous mutations affecting the telomerase reverse transcriptase subunit (*hTERT*) cause defects in stem cell proliferation, organ regeneration and incidence of cancer in humans. Patients with telomerase dysfunction including DKC, frequently develop aplastic anemia whereas telomerase‐null murine models display only modest hematopoietic defects.

Early models of DKC (hypomorphic DKC1) display a DKC‐like phenotype reflected in the increased evidence of tumors in the mammary glands and lungs (not in gut and skin as in human DKC patients), splenomegaly, dyskeratosis of the skin, anemia, yet in the early generations there is no obvious telomerase dysfunction 43. Similar findings have been reported for *Tert^‐/‐^* and *Tr^‐/‐^* early generations where classical degeneration phenotypes and telomere dysfunction could only be achieved after successive mattings which result in substantial erosion of telomeres and fusion and loss of chromosomes 44, 45. These observations are consistent with the probability that mouse telomeres are too long to erode sufficiently in a single mouse lifespan to generate all the DKC symptoms observed in humans. Deletion of the RNA template subunit (*mTERC*) in mice that already have short telomeres is a more effective means of replicating the DKC phenotype. Similarly, double knockout of *Pot1b* (an ortholog of the “protection of telomeres protein” encoding gene) and *Terc* results in enhanced telomere degradation (rather than progressive telomere shortening) and results in premature death, BMFS, significant anemia, leukopenia and thrombocytopenia. That such double knockout is necessary to create a DKC‐like phenotype calls into question once more the validity of murine models of human disease. Further, the question of how stem cell failure occurs at all in these models also arises. Loss or functional failure of HSC clearly occurs after significant erosion of telomeric DNA but whether this is due to induction of senescence by critically short telomeres is not yet clear. Other mechanisms to account for the dysfunctional HSC of DKC patients have been proposed such as defects in ribosome biogenesis leading to defects in the processing of 18s rRNA 65. This process has been modeled in zebrafish by knockdown of the gene *Nop10* and this model does show enhanced HSC apoptosis but to our knowledge; however this has not been attempted in mice.

A further complication with disease modeling of DKC using targeted gene knockdown in mice is the nature of human mutations. In most documented cases to date, partial loss of function as well as haploinsufficiency (for example *DKC1* in de novo DKC) and in some cases dominant negative mutations (for example *TINF2* in autosomal dominant DKC) have been reported in addition to loss of function mutations. Whilst, loss of function mutations can be modeled with targeted gene approaches in mice, partial loss of function and haploinsufficiency are difficult to mimic unless the human mutation is introduced into the mouse germ line or ESC using the most recent gene editing technique (for example Crispr/Cas9 method). This however can be easily superseded by the iPSC disease modeling approach which enables the assessment of human mutations in a dish by reprogramming of patient specific somatic cells.

The severity of the mutations is also an important aspect which cannot always be matched between mouse models and human patients. This is best exemplified by a rare and severe form of DKC, Hoyeraal‐Hreidarsson syndrome, which is caused by mutations in a subset of genes, including *DKC1, TINF2, TPP1* and *RTEL1* and manifests early in childhood. Human patients with two mutations in the *TPP1* gene (single amino acid deletion + missense mutation) require regular platelet and red blood cell transfusions for bone marrow failure 66; however homozygous mouse mutants of *Tpp1* were embryonic lethal 67, suggesting that the “blunt” loss of function created by targeted gene knock‐out in mice enables creation of much more severe phenotypes compares to human, but also result in loss of viability preventing further disease modeling. This also raises the intriguing question as to why the mouse homozygous mutants are lethal if telomeres in mice are much longer than humans, which suggests that the simple view of telomere length in stem cell renewal is not as important as telomere protection from degradation which is essential for maintaining the ends of chromosomes and genomic stability in proliferating stem and progenitor cells.

### Diamond‐Blackfan Anemia

Mutations in ribosomal protein genes that encode structural components of the ribosome responsible for the correct assembly of the ribosomal subunits are associated with the abnormal pre‐rRNA maturation patterns in DBA patients 68. *RPS19* gene mutations are found in 25% of DBA patients 69. Mutations resulting in haploinsufficiency or loss‐of‐function in all genes identified so far include missense mutations, nonsense mutations, splice mutations, insertions, deletions, and rearrangements 5. These mutations prevent the assembly of ribosomal protein to form preribosomal particles, which in turn activates nucleolar stress pathways that are at the center of the pathophysiology of DBA. Some of the mutations in *RPS19* can affect the synthesis of the ribosomal protein by altering transcription, splicing, or translation 70, 71, 72. RPS19 function is essential for correct processing within ITS1 and subsequent maturation of the 3′ end of 18S rRNA. Inactivation of both copies of *RPS19* genes in *Saccharomyces cerevisiae* yeast leads to complete arrest of small ribosomal subunit synthesis 73. Accumulation of large amounts of 21S pre‐rRNA was detected in DBA patients carrying *RPS19* mutations. This defect in pre‐RNA maturation was also observed in CD34− hematopoietic precursors, skin fibroblasts, and lymphoblastoid cell lines. As shown in Table 2, *Rsp19* ^‐/‐^ homozygous mice are embryonic lethal, whilst heterozygous mice either are normal or show a mild macrocytic anemia (depending on the nature of targeted gene event), indicating that these models are unable to truly recapitulate the *RPS19* haploinsufficiency described in the human DBA patients. Mice heterozygous for missense mutations of *Rsp19* and for a similar ribosomal component *Rsp20* show mild macrocytic anemia indicating possible HSC dysfunction. Other models including antisense oligonucleotide knockdown of *Rpl11* in Zebrafish show significant effects on early hematopoietic development and defects in adult hematopoiesis such as reduced formation and maturation of erythroid cells which is similar to the phenotype of DBA. Another informative model is the conditional *Rsp6* mouse where homozygous deletions of *Rsp6* were achieved through CD4‐driven Cre recombinase resulting in abrogation of T cell development. In contrast to homozygous deletions, *Rps6* haploinsufficiency although it did not impact T cell maturation, it affected their proliferation. Together these data suggest that blood cells with *Rsp6* haploinsufficiency may cope with ribosome synthesis under low cell proliferation; however this is severely compromised in tissues characterized by rapid proliferation (such as the hematopoietic system).

**Table 2:** Summary of key murine models of inherited bone marrow failure syndrome

**Disease phenotype/Gene name**	**Affected systems**	**References**
FA/*Fanca*	Homozygotes displayed FA‐like phenotypes including growth retardation, microphthalmia, craniofacial malformations and hypogonadism. Homozygous females demonstrate premature reproductive senescence and an increased incidence of ovarian cysts. Homozygous males exhibit an elevated frequency of mis‐paired meiotic chromosomes and increased apoptosis in germ cells, implicating a role for Fanca in meiotic recombination. *Fancc^*‐/‐*^ Fanca^*‐/‐*^* display the same phenotype as the single mutants suggesting that these two genes are epistatic.	28, 29
FA/*Fancc*	Homozygotes do not show developmental abnormalities or hematological defects till 9‐12 months of age. Male and female mutant mice have reduced numbers of germ cells and females have markedly impaired fertility. The CFC capacity of hematopoietic progenitors is abnormal and the cells are hypersensitive to gamma‐interferon. *Fancc^*‐/‐*^Tert^*‐/‐*^* double mutant mice have exacerbate telomere attrition when murine bone marrow cells experience high cell turnover after serial transplantation and increase in the incidence of telomere sister chromatid exchange. *Fancc^*‐/‐*^Fancg^*‐/‐*^* double‐mutant mice develop spontaneous hematologic sequelae, including bone marrow failure, acute myeloid leukemia, myelodysplasia and complex random chromosomal abnormalities.	30, 31
FA/*Fancd1*	Homozygous null mutants are embryonic lethal with abnormalities including growth retardation, neural tube defects, and mesoderm abnormalities; conditional mutations cause genetic instability and enhanced tumor formation; mutants with truncated BRCA2 protein survive, are small, infertile, show improper tissue differentiation and develop lymphomas and carcinomas	32
FA/*Fancd2*	Homozygous mutant mice exhibit meiotic defects and germ cell loss. In addition, mutant mice display perinatal lethality, susceptibility to epithelial cancer and microphthalmia. Homozygous mice have smaller hematopoietic stem cell pool and reduced lymphoid progenitor frequency. *Fancd2^*‐/‐*^Aldh2^*‐/‐*^* double homozygous mice are unusually sensitive to ethanol exposure *in utero*, and ethanol consumption by postnatal double‐deficient mice rapidly precipitates bone marrow failure and spontaneously developed acute leukemia. Aged *Aldh2^*‐/‐*^Fancd2^*‐/‐*^* mutant mice which do not develop leukemia, spontaneously develop aplastic anemia, with concomitant accumulation of damaged DNA within the hematopoietic stem and progenitor cell pool.	[33‐35]
FA/*Fancg*	Females and males homozygous for targeted null mutations exhibit hypogonadism and reduced fertility. Cytogenetic analysis shows somatic chromosome aberrations occurrence at a higher spontaneous rate. Cells are also more sensitive to mitomycin C.	36
FA/*Fanci*	These mice show craniofacial, vision, and eye abnormalities.	37
FA/*Fancn*	Homozygotes display embryonic lethality with impaired inner cell mass proliferation, impaired gastrulation, absence of the amnion, somites and tail bud, and general improper organogenesis.	38
FA/*Fancm*	Homozygotes exhibit reduced female transmission, hypogonadism, premature death and increased incidence of tumors.	39
FA/*Fancp*	Homozygotes display exhibit preweaning lethality, reduced fertility, abnormal eye morphology, abnormal skeletal morphology, hydrocephalus, chromosomal instability, early cellular replicative senescence and abnormal lymphopoeisis. Mutant mice are characterized by blood cytopenia, premature senescence, accumulation of damaged chromosomes and hypersensitivity to DNA cross linking agents.	40
FA/*Fanco*	Mice homozygous for a null mutation display embryonic lethality. Mice carrying a null and a hypomorphic allele have partial penetrance of male and female infertility due to defects in meiosis.	41
FA/*Fancs*	Homozygous null mutants are embryonic lethal with abnormalities including growth retardation, neural tube defects, and mesoderm abnormalities; conditional mutations cause genetic instability and enhanced tumor formation; mutants with truncated BRCA1 protein survive, have a kinky tail, pigmentation anomalies, male infertility and increased tumor incidence.	42
DKC/*Dkc1*	Early generation male mice hemizygous for a hypomorphic allele exhibit bone marrow failure, dyskeratosis, extramedullary hematopoieis, splenomegaly, lung and kidney abnormalities, increased tumor incidence, altered ribosome function. Decreased telomere length is noted only in later generations.	43
DKC/*Tert*	In spite of impaired telomerase function, homozygous mutant mice are overtly normal in early generations. Impaired fertility has been reported in later generations for homozygotes of at least one knockout allele. Homozygous Tert mice display short dysfunctional telomeres and sustained increased DNA damage signaling and classical degenerative phenotypes upon successive generational mattings and advancing age.	44
DKC/*Tr*	Early generation mice homozygous for a null allele have intact telomeres and appear grossly unaffected and healthy, whereas late generation mutants exhibit premature death, shortened and dysfunctional telomeres, apoptotic and proliferative defects, infertility, and multi‐organ degenerative decline. Late‐generation animals exhibit defective spermatogenesis, with increased programmed cell death (apoptosis) and decreased proliferation in the testis. Proliferative capacity of hematopoietic cells in the bone marrow and spleen is also compromised. These progressively adverse effects coincide with substantial erosion of telomeres and fusion and loss of chromosomes.	45
DKC/*Tinf2*	Targeted disruption of this gene results in embryonic lethality prior to E7.5 through a mechanism that is independent of telomerase function. Second and third generation heterozygotes develop mild pancytopenia, consistent with hematopoietic dysfunction in DKC, as well as diminished fecundity.	46
DKC/*Rtel1*	Homozygous null mice display embryonic lethality with abnormal development of the neural tube, brain, heart, vasculature, placenta, and allantois and chromosomal abnormalities in differentiating cells.	47
DKC/*Ctc1*	Mice homozygous for a targeted allele exhibit defective telomere replication that leads to stem cell exhaustion, bone marrow failure and premature death.	48
DBA/*Rps6*	Conditional Rps6 mice using CD4‐Cre abolishes T cell development.	49
DBA/*Rps7*	R*ps7* disruption results in decreased body size, abnormal skeletal morphology, mid‐ventral white spotting, and eye malformations. *Rps7* mutants display overt malformations of the developing central nervous system and deficits in working memory; however they do not show anemia or hyperpigmentation.	50
DBA/*Rps19*	Homozygous null embryos die prior to the formation of a blastocyst. Mice heterozygous for some point mutations show pigment defects affecting the feet and tail. However the heterozygotes show a normal development of the hematopoietic system. Heterozygous missense mutations of *Rps19* show a mild macrocytic anemia reflecting the fact that mutations causes a hypomorphic allele rather than true happloinsufficiency.	51
DBA/*Rps20*	Heterozygous missense mutations of Rps20 show a mild macrocytic anemia reflecting the fact that mutations causes a hypomorphic allele rather than true happloinsufficiency.	52
SDS/*Sbds*	Loss of *Sbds* gene results in early embryonic lethality, with homozygotes showing histological abnormalities of the liver and accumulation of free cytoplasmic 40 S and 60 S subunits. Heterozygotes have a normal phenotype.	53
CAMT/*Mpl*	Mice homozygous for targeted mutations at this locus are unable to produce normal numbers of megakaryocytes and platelets and display HSC deficiencies that are not limited to megakaryocytic lineages. These mice also have increased concentrations of circulating TPO.	54
SCN/*Ela2*	Homozygotes for a null allele show impaired neutrophil physiology, susceptibility to Gram (‐) bacterial infection, reduced sensitivity to xenobiotics and abnormal local Schwartzman responses. Homozygotes for a knock‐in allele show susceptibility to fungal infection and resistance to endotoxic shock. Heterozygous mice do not show neutropenia.	55
SCN/*HAX1*	Mice homozygous for deletion of this gene fail to survive beyond 14 weeks of age. Apoptosis of neurons in the striatum and cerebellum occurs as does loss of lymphocytes and neutrophils.	56
SCN/*Gfi1*	Homozygotes are severely neutropenic and accumulate immature monocytes in blood and bone marrow. Their myeloid precursors cannot differentiate into granulocytes upon stimulation with G‐CSF; however they can develop into macrophages. Conditional knockouts indicate defects in Th2 cell expansions and enhanced IFNγ production.	57
SCN/*Wasp*	Homozygous mutant females and hemizygous mutant males exhibit reduced numbers of peripheral blood lymphocytes and platelets, but increased numbers of neutrophils.	58
RS/*Hoxa11*	Homozygotes for targeted null mutations exhibit homeotic transformations affecting thoracic and sacral vertebrae, and forelimb defects. Mutants are sterile due to malformed vas deferens and cryptorchism in males, and defective uteri in females.	59

Abbreviations: CFC, colony‐forming cell; G‐CSF, granulocyte colony stimulating factor; FA, Fanconi anemia; HSCs, hematopoietic stem cells; IFNγ, interferon γ; TPO, thrombopoietin.

### Shwachman‐Bodian‐Diamond

Knockouts of two genes implicated in Shwachman‐Bodian‐Diamond syndrome (*Sbds)* and ribosomal protein 18 (*Rps18)* which are central to ribosome function are unsurprisingly embryonic lethal 74. Approximately 90% of SDS patients have mutations in the *SBDS* gene which shares 97% homology with an adjacent pseudogene, *SBDSP* which contains deletions and nucleotide changes preventing generation of a functional protein. 75% of patients with SDS have *SBDS* mutations which are acquired as result of gene conversion events from the pseudogene *SBDSP*. Patients homozygous for *SBDS* mutations have not been identified, suggesting that complete loss of SBDS is likely to be lethal in humans. This is corroborated by animal studies which have shown that loss of *Sbds* gene in mice results in early embryonic lethality (Table 2), whilst heterozygous mice are completely normal, indicating once more the inability of heterozygous mouse mutants to mimic the precise nature of human *SBDS* mutations. While studies in yeast have clearly shown disruption of ribosome biogenesis as result of SBDS dysfunction, how this leads to a specific phenotype in the bone marrow of SDS patients remains to be identified.

### Severe Congenital Neutropenia

SCN is characterized by a defective neutrophil maturation leading to severe infections. Human SCN‐associated mutations in *ELA2, GFI1,* and *WASP* appear not to be functionally null in humans and this creates some difficulty when comparing the phenotype of SCN in humans and animal models. A clear example of this is provided by the *Gfi1* knockout model which shows a much more severe phenotype compared to patients with SCN 75. Similarly *Ela2^‐/‐^* mice display impaired neutrophil function, consistent with a role for elastase 2 in bacterial killing; however heterozygous *Ela2* mice do not show neutropenia. This suggests that to date there is no suitable animal model for mimicking *ELA2* causing SCN in man. For *HAX1*, all three known human mutations lead to premature stop codons during the translation stage and therefore it was hoped that *Hax1^‐/‐^* knockout models would mimic the disease phenotype. Unfortunately, *Hax1^‐/‐^* mice fail to survive past 14 weeks of age, thus preventing functional studies of this gene in adulthood.

## The Advantages of In Vitro Modeling

From the range of studies described so far, it should be clear that animal models of BMFS are at best only approximations of the etiological mechanisms in humans. Thus, there is a clear and largely unmet need to create alternative disease models to further our mechanistic understanding of BMFS and to explore management approaches. Despite huge efforts, the last decades have witnessed an alarming failure to develop new treatments. At the center of this dilemma is the fact that animal disease models do not mirror human disease, thus the drug is either ineffective or leads to unacceptable toxicities in humans. Drug discovery based on affected human models would yield superior results, but unfortunately affected tissues are often accessible only on deceased patients. Such tissues are unsuitable for testing drug toxicity and efficacy as they reflect end stages of the disease. This is particularly true for inherited BMFS since ex vivo expansion of HSC from aplastic bone marrow is challenging 76.

The advent of human pluripotent stem cell technology may offer a solution to these problems. Human embryonic stem cells (hESCs) and more latterly, induced pluripotent stem cells (iPSCs), are capable of indefinite in vitro expansion while retaining the ability to differentiate into cell types characteristics of most tissues found in the developing embryo. Reprogramming somatic cells to pluripotency by ectopic expression of four transcription factors expressed by ESC was first reported in 2006 77. The products of such reprogramming were termed “induced pluripotent stem cells” or iPSC by their originator, Shinya Yamanaka. In the following year, human iPSC (hiPSCs) were reported by Yamanaka's and another group 78, 79. The pluripotency of iPSCs confers properties on these cells particularly interesting for clinical research since iPSCs can be patient‐specific and disease‐specific, thus providing a promising platform for investigating the pathophysiology of a specific disease and testing the effectiveness and toxicity of drugs in the cell of interest. Despite the need to overcome some potential hurdles such as the acquisition of de novo genetic alterations during reprograming, possible retention of a small number epigenetic marks present in the somatic parent cells and aberrant DNA methylation acquired during the reprogramming process, which may affect the cell differentiation capabilities, there is an increasing awareness of the value of iPSC based disease modeling as a tool to further drug discovery 80, 81, 82, 83.

Differentiation of pluripotent stem cells toward patient specific hematopoietic cell types is the basis of modeling inherited BMFS. However, the major challenge is the generation of fully functional HSCs. Several studies have described the generation of hematopoietic progenitors, but on further differentiation in both in vitro and in vivo experimental models, such progenitors show a preference toward myeloid differentiation so high long term engraftment efficiency and multi‐lineage differentiation of functional HSC in the bone marrow of immune compromised mice remains challenging 84, 85, 86, 87, 88, 89, 90. Several patient specific hiPSCs lines from patients with inherited BMFS have now been established. To maintain clarity with respect to our discussion of animal models we will describe hiPSCs based models of BMFS in the same order before discussing additional in vitro modeling attempts.

### Fanconi Anemia

The processes of reprogramming needed to generate hiPSCs is dependent on enhanced cellular proliferation during the initial stages of reprogramming which requires the activation of DNA repair pathways to ensure maintenance of genomic stability. Thus, it is quite difficult to derive hiPSCs from somatic cells of patients suffering from DNA repair disorders, which renders the development of a hiPSCs based model of FA difficult to achieve. Initial studies suggested that reprogramming of FA patient specific hiPSCs was only possible after genetic complementation of the donor fibroblasts prior to reprogramming or when reprogramming was performed under hypoxic conditions 91, 92. The increased incidence of unrepaired DNA double strand breaks in FA patients was thought to contribute to this. An alternative approach to modeling FA is to knockdown the expression of FA genes in otherwise healthy hESCs and some data were generated by Tulpule et al. by RNA interference mediated knockdown of *FANCD2* and *FANCA* 93. Differentiation of hESCs subjected to this approach yielded hematopoietic progenitors with reduced ability to mature into functional CD45+ cells. Furthermore, these FA‐deficient hematopoietic progenitors displayed an abnormal transition of embryonic to adult globin expression, indicating an impaired hematopoietic development with a bias toward primitive populations. Yung and coworkers succeeded to reprogram FA‐C fibroblasts under normoxic conditions, although the reprogramming efficiency was much lower compared to fibroblasts taken from unaffected controls 94. These hiPSCs showed frequent chromosomal abnormalities and were unable to generate bona‐fide teratomae with contribution to cell types representative of all three embryonic germ layers. Despite this, the FA‐C hiPSCs were able to differentiate into hematopoietic progenitors albeit ones with significantly reduced clonogenicity and enhanced levels of apoptosis. A greater percentage of the phenotypic hematopoietic progenitors from FA patient specific iPSC were in S‐phase of the cell cycle indicating a higher rate of proliferation and the possibility that this is a compensatory response to the higher levels of apoptosis. Replenishment of hematopoietic progenitors at this higher rate contributes to the development of more chromosomal abnormalities and replicative exhaustion of the stem cell population. Whilst the increased apoptosis of hematopoietic progenitors and accumulation of genomic instabilities were features already described in mouse models, the role of FA pathway during somatic cell‐induced reprogramming and the availability of FA‐specific cells for drug discovery are novel aspects which could only be achieved through the hiPSCs disease modeling approach. For regenerative medicine, caution should be exercised to obtain patient specific cells as early as possible and to ensure they are genetically complemented with the functional alleles before the reprogramming and differentiation process in order to maintain genomic stability.

### Dyskeratosis Congenital

hiPSCs have been derived from DKC patients with mutations in *DKC1, TERC, TERT,* and *TCAB1* which display normal karyotypes and hallmarks of pluripotency as described earlier 95, 96, 97, 98. Since telomerase activity is normally increased during the reprogramming process, by upregulation of the catalytic subunit hTERT, one might expect that DKC specific iPSC would be less useful as disease models. This is probably true for DKC cases involving mutations in the RNA template component (TERC) since hTERT reactivation appears to be sufficient to overcome the reduction in telomerase activity resulting from the *TERC* mutations. However, attempted upregulation of an h*TERT* gene carrying heterozygous mutations merely produces a dysfunctional hTERT protein, which cannot function effectively within the telomerase holoenzyme complex. For this reason, hiPSCs lines derived from patients with such h*TERT* mutations display shortened telomeres following extended culture which ultimately prevents their self‐renewal. This phenotype is more pronounced in hiPSCs lines derived from patients with X‐linked DKC where the mutation of *DKC1* blocks telomerase assembly and disrupts telomere elongation 95. The severity of the telomerase dysfunction depends upon the precise nature of the *DKC1* mutation; hiPSCs lines carrying Q31E and ΔL37 mutations maintained the same telomere length as the parental fibroblasts; however the A353V mutant DKC‐ hiPSCs presented shorter telomeres compared to the parental fibroblasts suggesting that this A353V mutation has a more severe effect on the telomere maintenance process.

### Diamond‐Blackfan Anemia

DBA‐hiPSCs have been generated from patients with mutations in *RPS19* and *RPL5* genes 99. These DBA‐hiPSCs lines exhibited defective assembly of ribosomal subunits and production of ribosomal RNA as well as impaired generation of hematopoietic progenitors with erythroid lineages being the most affected. These features recapitulate the phenotype observed in DBA patients, thus providing an excellent tool to investigate DBA. Genetic correction of the affected genes restored the expression of deficient ribosomal proteins resulting in an increase of the ribosome biogenesis, normal generation of hematopoietic progenitors and erythroid cells. Using patient specific lines and hematopoietic differentiation as a model, the same authors described for the first time the dysregulation of noncanonical TGFβ signaling pathway mediated by p‐JNK which may be the underlying cause for abnormal hematopoiesis in DBA‐hiPSCs, for TGFβ signaling is inhibitory to hematopoietic commitment 100. These studies clearly indicate that patient specific hiPSCs can provide novel insights into the disease pathogenesis as well as a platform for regulating critical pathways involved in hematopoiesis and erythroid differentiation via the use of small molecules.

### Shwachman‐Bodian‐Diamond

hiPSCs have been generated from patients with reduced *SBDS* expression which display dysfunctional ribosome assembly 101. Upon differentiation to pancreatic cell lineages, the SDS‐hiPSCs and hESCs showed increased cell death and impaired organization of the acinar‐like structures, which led to progressive loss of exocrine tissue. SDS‐hiPSCs were able to differentiate to mesodermal lineages; however a reduced percentage of hematopoietic cells and colony forming potential was noted together with increased levels of proteases in culture supernatant. The residual *SBDS* expression in some patient specific lines led to absence of the pancreatic phenotype and presence of hematopoietic defects only, similar to previous clinical observations in SDS patients, thus further corroborating the genotype‐phenotype correlation. Despite this observed variability, both the pancreatic and/or hematopoietic phenotype could be rescued either by the forced expression of the *SBDS* transgene or by the use of protease inhibitors in the culture media, thus providing a clear example of drug‐reversible phenotype using the hiPSC model for these patients and providing novel therapeutic insights.

### Severe Congenital Neutropenia

Several groups have reported the generation of patient specific hiPSCs carrying mutations in genes involved in SCN such as *ELA2* and *HAX1* 102, 103. Upon differentiation to neutrophils using a defined feeder free method, SCN‐hiPSCs lines show developmental arrest at the myeloid progenitor stage resulting in decreased pool of myeloid progenitors, impaired response to G‐CSF stimuli as well as increased levels of apoptosis in neutrophils, reminiscent of abnormal granulopoiesis observed in SCN patients. This phenotype could be rescued by retroviral transduction of *HAX1* and *ELA2* in the respective isogenic hiPSCs lines. In a recent study, Nayak et al. identified the mislocalization of the *ELA2* gene encoding protein neutrophil elastase (NE) as the inductor of the UPR/ER stress, dysfunctional differentiation and apoptosis and demonstrated that the SCN phenotype could be corrected by addition of the NE inhibitor called sivelestat to the culture media of the hiPSC‐derived myeloid progenitors. Together these studies indicate that SCN‐hiPSCs provide an excellent platform for high‐throughput screening of drugs to reverse various congenital neutrophil disorders as well as better understanding of disease pathogenesis which can be clinically exploited to achieve therapeutic responses using lower doses of G‐CSF combined with targeting to correct NE mislocalization.

Recent progress in the field of genome editing is giving iPSC technology a solid framework for disease modeling and for future clinical therapies. Previous gene therapies carried out via transient or constitutive expression of transgenes were undermined by the possibility of transgene integration into loci that conferred uncontrolled cellular proliferation resulting in tumorigenesis. To date the development of robust and efficient human genome engineering tools including zinc‐finger nucleases (ZFNs), transcription activator‐like effector nuclease (TALEN) and Crispr‐Cas9 systems which permit in situ gene editing with reduced risk of off‐site mutagenesis has propelled forward the genome engineering approaches 104. In particular, the Crispr/Cas9 system stands out from the other gene editing tools for its precision, versatility and simplicity. Early applications of in situ gene editing approaches involved introduction of specific gene mutations for disease modeling studies in various species ranging from fruit flies to Man 105, 106, 107. Currently genome editing tools are being used to correct genetic defect in iPSC harboring disease‐causing mutations providing insights on the pathophysiology of hematological disorders (such as β‐thalassemia or hemophilia) and confirming disease causality of the corrected genetic defect 108, 109, 110, 111, 112.

To date, a handful of studies have reported the successful use of genome editing tools for the study of BMFS. For example, ZFN mediated disruption of the *FANCA* gene in wildtype hESC to create a FA disease model and restoration of specific mutations in the *FANCC* gene in wild type fibroblasts via the Crispr/Cas9 system have been reported 113, 114. Recently, Bluteau et al. inactivated the *REV7* using the Crispr/Cas9 approach and showed that this resulted in increased cellular hypersensitivity to DNA interstrand cross‐link drugs (ICL) as well as an impaired ability of the mouse hematopoietic progenitor cells to form hematopoietic colonies in Colony‐Forming Unit (CFU) assay, resulting in characterization of a new gene in the FA pathway 116. Combination of genome editing tools with the iPSC technology has the potential to result in generation of disease free iPSC derived hematopoetic cells that can be used in potential cell based therapies. Rio et al. reported in 2014 the successful phenotypic correction of *FANCA* defective fibroblasts and later generation of disease‐free iPSC which upon hematopoetic differentiation could generate similar number of hematopoietic colonies compared to healthy cord blood progenitor cells 117. Likewise, the disease‐free iPSC‐derived hematopoetic progenitors proved to be resistant to exposure to drugs that cause interstrand cross‐links in DNA thus demonstrating the reversal of disease phenotype using novel and safe gene editing tools.

Application of iPSC technology as a platform for gene correction and drug screening was also reported for DKC 117. By using CRISP/Cas9 genome editing system Woo et al. reported the genetic correction of mutation in *DKC1* in patient specific IPSC as well as introduction of *DKC1* mutation in wild type iPSC. As expected, the corrected *DKC1*‐iPSC showed high telomerase activity, whereas *DKC1* mutant‐ iPSC showed lower telomerase activity than wildtype controls. Interestingly, this study also provided data that supported the link between *DKC1* and Wnt signaling pathway by showing restoration of telomere length, telomere capping and reduction of p53BP1 telomere foci upon application of the Wnt agonist CHIR99021 in DKC‐iPSC, indicating that both gene editing and manipulation of signaling pathway provide potential avenues for treatment of BMFS.

To date, allogeneic HSC transplantation from HLA‐identical healthy donors remains the only curative therapy for BMFS patients. The possibility of performing gene correction approaches in patients HSCs together with an improved yield of HSC expansion so that sufficient numbers of cells for autologous transplantation can be achieved, offers a revolutionary prospect for the field of BMFS. This will require a reduction in off‐target mutagenesis; however with the pace the field is progressing, one would hope that this is within our reach very soon.

## Generation of In Vitro Cellular Models That Have Not Been Modeled in Animals

### CAMT‐hiPSC

CAMT is characterized by the loss of function or deletion of the thrombopoietin (TPO) receptor encoded by the *MPL* gene, resulting in loss of megakaryocytes (MK) in the bone marrow, severe thrombocytopenia and development of fatal BMF later in life. Hirata et al. investigated the mechanisms involved in the MPL signaling and the development of MK/Erythrocyte progenitor (MEP) by generating hiPSCs from CAMT patients carrying MPL mutations 118. The resultant CAMT‐hiPSCs failed to generate MKs and platelets after hematopoietic differentiation, thus recapitulating the typical feature of human disease: thrombocytopenia. Likewise, CAMT‐hiPSCs displayed impaired colony formation capability especially with regard to erythrocytes and megakaryocytes, demonstrating a critical role for MPL signaling in the formation of the common MEP progenitor which is able to generate both lineages. Retroviral transduction of MPL transgene in CAMT‐hiPSCs restored the differentiation potential of the CAMT to generate MK, platelets and erythrocytes to similar levels as in control hiPSCs. Interestingly, excessive expression of MPL transgene led to a dysregulation of thrombopoiesis and defective megakaryopoiesis suggesting that MPL signaling is finely regulated and levels of expression must fall within a precise expression window for normal development of thrombopoiesis. These insights gained through the hiPSC approach are particularly important for designing curative gene correction strategies which should aim to restore an appropriate level of MPL expression.

### MDS‐hiPSC

MDS is the most common form of primary BMF. The somatic loss of the long arm of chromosome 7 (del (7q) is one of the most characteristic chromosomal abnormalities in MDS. Recently, Kotani et al. derived hiPSCs clones that displayed normal karyotype as well as clones with del(7q) from MDS patients 119. Whereas normal isogenic hiPSCs were able to generate hematopoietic progenitors, MDS‐hiPSCs displayed a reduced hematopoietic differentiation potential and clonogenic capacity in all myeloid lineages. Furthermore, MDS‐hiPSCs showed an increase in cell death rate during differentiation. These observations are consistent with the phenotype observed in primary MDS cells. Interestingly, the authors observed a spontaneous dosage correction in one of the clones which acquired a 30 Mb region of a telomeric part of chromosome 7q (chr7q) and which upon differentiation showed a fully restored hematopoietic potential to a level similar to normal hiPSCs. This suggests that the defect responsible for the abnormal hematopoietic phenotype is localized in this region. Gene expression comparison between del(7q) MDS clones and chr7q dosage corrected clones allowed the authors to identify 4 haploinsuficient candidate genes whose forced expression could partially rescue the hematopoietic defects in del(7q) MDS iPSC. This study offers an interesting approach for functional mapping and identification of haplo‐insufficient genes involved in large‐scale chromosomal deletions‐associated disorders such as MDS using the hiPSC technology.

## Summary

**Figure 1:**
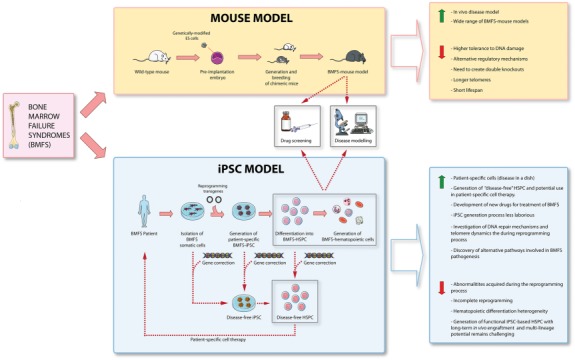
A schematic summary of advantages and disadvantages of animal and human iPSCs based disease modeling approaches. Abbreviations: HSPC, hematopoietic stem and progenitor cells; iPSCs, induced pluripotent stem cells.

In the last 20 years, great progress has been achieved in identifying new genetic causes/susceptibilities of inherited BMFS, which has resulted in better genotype‐phenotype correlations, improved diagnosis and clinical treatments. A large number of mouse models have been created enabling generation of insights into disease mechanisms and pathology. Nevertheless, the inability of gene knock‐ins/out to mimic the precise nature of human mutations and especially happloinsufficiency together with differences in DNA damage tolerance, telomere length and short life span have undermined their utility in mimicking the full spectrum of BMFS (Fig. 1). The advent of induced pluripotency enabling generation of patient specific hematopoietic cells carrying the genetic change responsible for the disease has revolutionized the field and has provided a functional platform for uncovering new signaling pathways and small molecules that can be explored therapeutically. The tremendous advances made in the genetic engineering field has enabled in situ gene corrections in patients fibroblasts, hiPSC and HSCs opening new avenues toward generation of autologous cell replacement therapies for BMFS. Several improvements need to be achieved toward the generation of long term reconstituting HSC from hiPSC, efficient ex vivo expansion of HSCs obtained from adult bone marrow and reducing the off‐site targets of in situ gene editing; nevertheless the fast pace of progress made in the field of stem cells and genetic engineering boasts for a bright and promising future for treatment of BMFS which could have not been anticipated at the beginning of this century!

## Author Contributions

S.H. and D.M.S.: Conception and design, manuscript writing, final approval of manuscript; G.E.K. and S.A.: manuscript writing, final approval of manuscript; S.S., L.A., and M.L.: Conception and design, manuscript writing, final approval of manuscript, fund raising.

## Disclosure of Potential Conflicts of Interest

The authors no potential conflicts of interest.
